# HaSAPPy: A tool for candidate identification in pooled forward genetic screens of haploid mammalian cells

**DOI:** 10.1371/journal.pcbi.1005950

**Published:** 2018-01-16

**Authors:** Giulio Di Minin, Andreas Postlmayr, Anton Wutz

**Affiliations:** Institute of Molecular Health Sciences, Department of Biology, Swiss Federal Institute of Technology ETH Hönggerberg, Zurich, Switzerland; Hebrew University of Jerusalem, ISRAEL

## Abstract

Haploid cells are increasingly used for screening of complex pathways in animal genomes. Hemizygous mutations introduced through viral insertional mutagenesis can be directly selected for phenotypic changes. Here we present HaSAPPy a tool for analysing sequencing datasets of screens using insertional mutations in large pools of haploid cells. Candidate gene prediction is implemented through identification of enrichment of insertional mutations after selection by simultaneously evaluating several parameters. We have developed HaSAPPy for analysis of genetic screens for silencing factors of X chromosome inactivation in haploid mouse embryonic stem cells. To benchmark the performance, we further analyse several datasets of genetic screens in human haploid cells for which candidates have been validated. Our results support the effective candidate prediction strategy of HaSAPPy. HaSAPPy is implemented in Python, licensed under the MIT license, and is available from https://github.com/gdiminin/HaSAPPy.

This is a *PLOS Computational Biology* Software paper.

## Introduction

Next generation sequencing (NGS) has facilitated the exploration of animal genomes in a number of areas including recent approaches for genetic screening. In mammals, screening of important biomedical pathways can be performed orders of magnitude faster in cell cultures than in the organism and at a fraction of the cost. Cell based screens have been performed using RNA interference [1], sequence specific nucleases [2], and mutagenesis of haploid cells. The latter strategy has been originally implemented in a haploid human leukemia cell line [3] and recently extended to haploid embryonic stem cells (ESCs). A number of successful screens illustrate the power of pooled mammalian haploid cell screening. Clinically relevant pathways [4,5] including pathogen [3,6–8] and toxin [9–12] mechanisms have been studied in human haploid cells. Haploid ESCs from mouse [13,14] and from human embryos [15] have been used to characterize mechanisms of drug action [13,14,16] and developmental questions [17,18].

In haploid cells mutations can be introduced in a hemizygous state by chemical mutagenesis [19], viral [13,18,20], and transposon vectors [16,17] ensuring that potential phenotypic changes become expressed. Several screens have focused on gene trap vectors, which are characterized by high mutagenicity. Thereby the identification of mutations is possible through cloning the genomic flanking regions of the insertion sites. Typically, cell pools containing tens of millions of viral insertions are subjected to selection for predefined phenotypic characteristics. Comparison of viral insertions before and after selection is used to detect candidate genes, whose mutations become enriched. Use of NGS techniques to simultaneously analyse millions of insertion sites in large cell pools without isolating clonal cell lines has facilitated comprehensive screening in mammalian cells [3].

Previously, computational methods for candidate identification in microbial genetic insertional screens [21] and mammalian haploid cell screens [22] have been developed. Large mammalian genomes pose challenges as a lower mutation density can be achieved and regional differences in chromatin packaging can bias the distribution of insertions. Here, we present HaSAPPy (Haploid Screen Analysis Package in Python) for computational candidate identification. HaSAPPy analyses NGS datasets to reconstruct viral insertions in control and selected cell pools, estimates the enrichment of disruptive mutations, and the ratio of disruptive over neutral mutations for each gene. From the fold enrichment of these three parameters during selection candidate genes are identified as outliers from the distribution of all genes. We demonstrate that this strategy is effective by benchmarking candidate prediction from published datasets from screens aimed at identifying genes required for *Xist*-mediated gene repression [18] and for viral entry [8,20] in mouse and human haploid cells.

## Design and implementation

**Fig 1:**
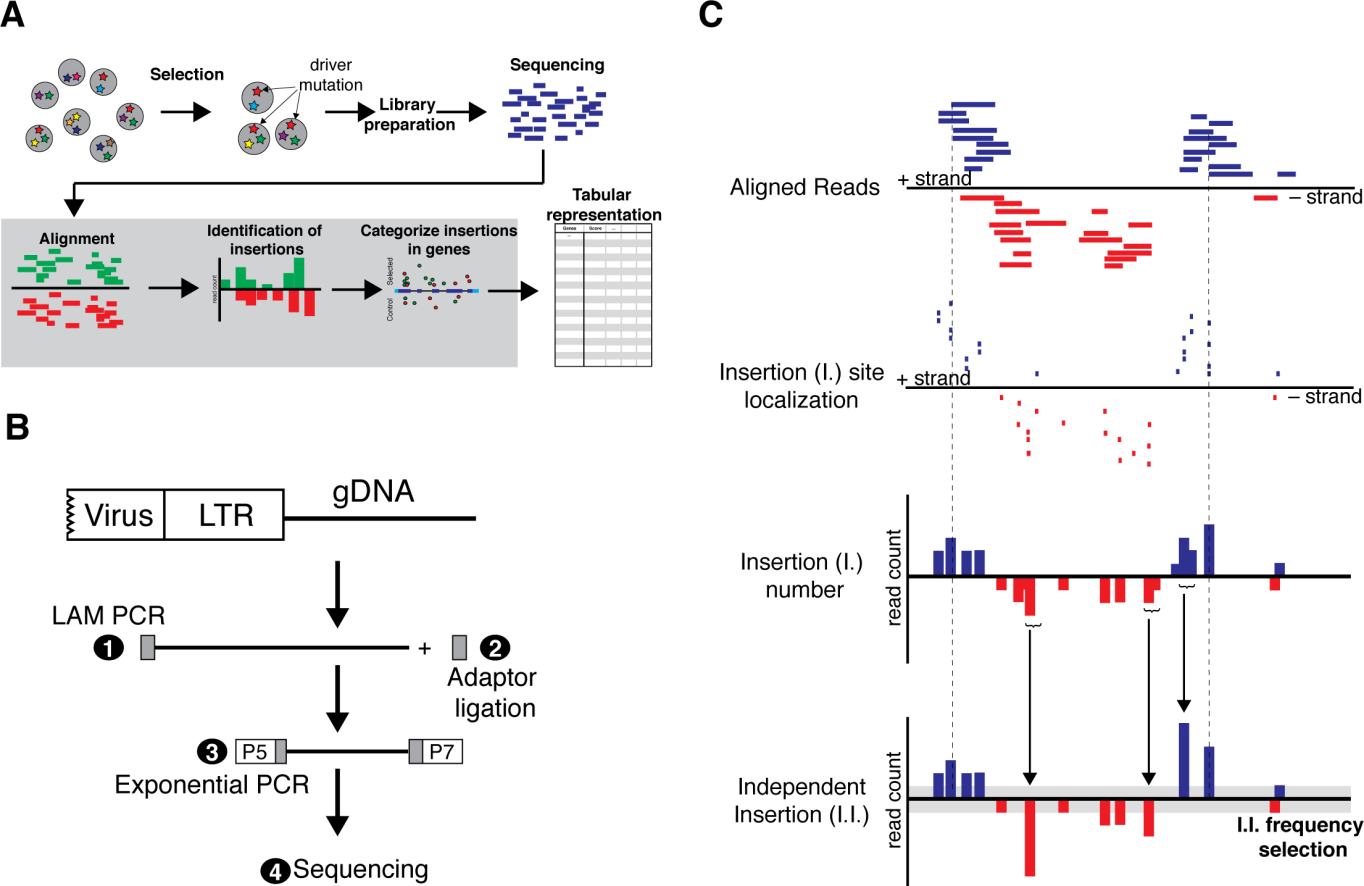
Overview of insertional mutagenesis screening in haploid cells. (**A**) Mutations are introduced into haploid cells by gene-trap vectors and subsequently selected for a desired phenotype for enriching driver mutations. Genomic regions flanking viral insertion sites are amplified and NGS libraries are prepared for sequencing. Subsequently, reads are aligned to the reference genome, insertions are reconstructed and localized in genes. Parameters for candidate selection are output in a tabular format. (**B**) Experimental strategy for NGS sequencing genomic regions flanking viral insertions. Linear amplification (LAM PCR) of genomic flanking sequences using a specific primer in the virus vector is performed (1). LAM PCR products are purified and a single stranded adaptor is ligated at the 3’end (2). NGS libraries are amplified by exponential PCR using primers at the end of the viral LTR and in the adaptor (3), and subsequently sequenced (4). P5, P7 represent Illumina NGS adaptors. (**C**) For reconstruction of virus insertion events the mapping of the first base of each read is assumed to represent the genomic position of the insertion event (I.). Read alignments are collapsed in a genomic window in a strand specific manner into the position of an independent insertion (I.I.), which is chosen as the position with the highest initial read count. The cumulative read count is reported and only insertions that satisfy a read count threshold (grey) are considered in the analysis.

HaSAPPy uses NGS datasets for predicting candidates from insertional mutagenesis screens in haploid cells based on selection for specific phenotypes including genetically encoded reporters, cell survival, and physical isolation of cells (Fig 1A). Gene-trap insertion sites are identified by reads starting with the first base of the genomic sequence flanking the vector insertion [23] (Fig 1B). Reads are preprocessed for eliminating regions with consistently low base quality and maintaining a maximum of sequence information. Subsequently, adaptor removal is performed and reads that become shorter than a threshold (default 26nt) are discarded. HaSAPPy has been preconfigured with three read mappers including the Burrows-Wheeler transform based Bowtie2 [24], and nvBowtie (https://github.com/NVlabs/nvbio/tree/master/nvBowtie), and the hash table index structure based NextGenMap [25] using a test suite [26]. HaSAPPy also accepts pre-aligned datasets in Sequence Alignment/Map (SAM) format and a threshold for alignment quality (MAPQ).

### Reconstruction of virus insertions

**Fig 2:**
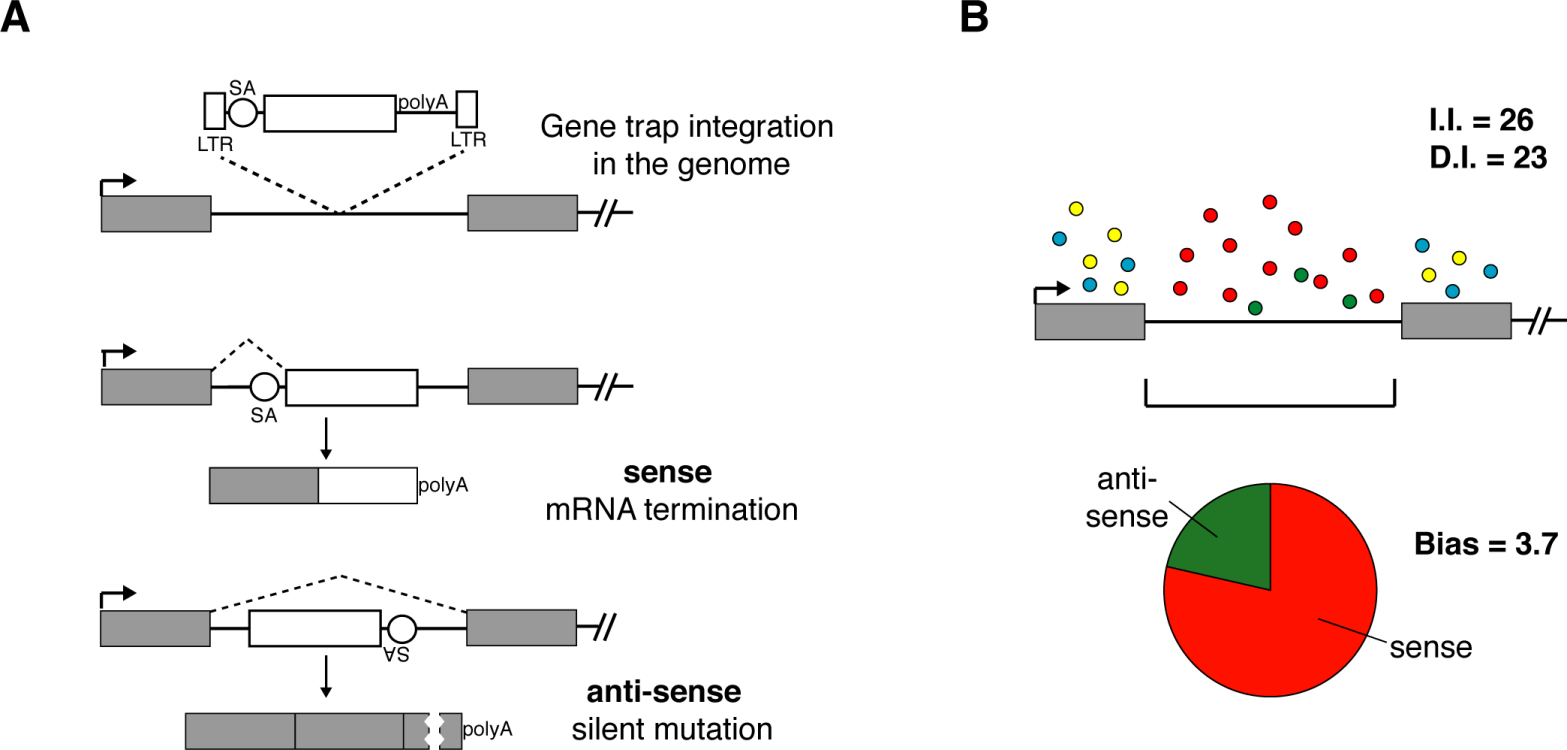
Parameters for measuring selection of viral insertions. (**A**) Disruptive insertions (D.I.) are calculated as the sum of exonic I.I. and intronic I.I. for which the splice acceptor of the gene-trap is oriented in the direction of transcription of a gene. In this orientation the gene-trap vector will truncate the gene and can be predicted to have a strong mutagenic effect. In reverse orientation the splice acceptor is not functional rendering intronic anti-sense insertions neutral. (**B**) A Bias value is calculated as the ratio of sense (red) over anti-sense (green) intronic gene-trap insertions within one gene measuring the excess of disruptive over neutral mutations. Bias can therefore provide evidence for selection independent of the number of I.I. and D.I.. Exonic mutations are considered mutagenic in sense (blue) and anti-sense (yellow) orientation and are excluded from Bias calculation.

Unequal amplification during NGS library preparation makes read numbers ineffective for estimating selection. We reconstruct independent insertions (I.I.) from the start positions of read alignments in a strand specific manner (Fig 1C). To avoid that sequence errors on read ends lead to multiple counting of insertions, reads within a genomic window on the same strand are attributed to the same virus insertion (Fig 1C) and collapsed onto a single I.I. at the position with the highest initial read count. A single I.I. per genomic position guarantees that all insertions are independent. Genome annotation from Refseq Genes or ENSEMBL transcripts, which is available through the UCSC genome browser Tables interface (http://genome.ucsc.edu/cgi-bin/hgTables?command=start), is used for defining genomic intervals of genes [27] that cover all transcript variants listed for a unique gene name. To further evaluate the mutagenic effect of insertions two sub-regions ‘Exon_specific’ and ‘Introns’ are considered. Counts are obtained by iteration over I.I. from each dataset for all genes. Insertion counts for introns are obtained in an orientation specific manner for assessing the trapping function of the splice acceptor of the gene-trap (Fig 2A). Disrupting insertions (D.I.) are calculated as the sum of exonic I.I., and intronic I.I. with the splice acceptor aligned with transcription. A bias is calculated as the ratio of intronic insertions in sense over anti-sense orientation for each gene (Fig 2B). I.I., D.I., and Bias are the basic parameters for estimating selection of genes in a screen.

### Candidate identification

HaSAPPy allows the analysis of multiple experiments against a single control, whereby each dataset can contain multiple replicates. For predicting candidates we implement a method that evaluates multiple parameters in parallel. Each gene is represented by a vector composed of the fold enrichment of I.I., D.I., and Bias in selected relative to control datasets. The majority of genes, for which mutations are not selected, clusters in this vector space. Selection is detected as the distance of a gene from this cluster by divergence of one or more parameters using the Local Outlier Factor (LOF) [28]. Candidates are then ranked by evidence for selection by sorting score factors in decreasing order.

### Implementation

HaSAPPy is written in Python and depends on HTSeq [27] for handling sequence files and genomic coordinates, and pandas, matplotlib, numpy, scipy, sklearn, and xlsxwriter for data analysis and output. Parameters of the analysis including table layout and graphics are specified in a text file that is used to instantiate HaSAPPy. Sorting, filtering, and visualization of insertions over candidate gene regions in SVG format is supported (S1 Fig).

## Results

**Fig 3:**
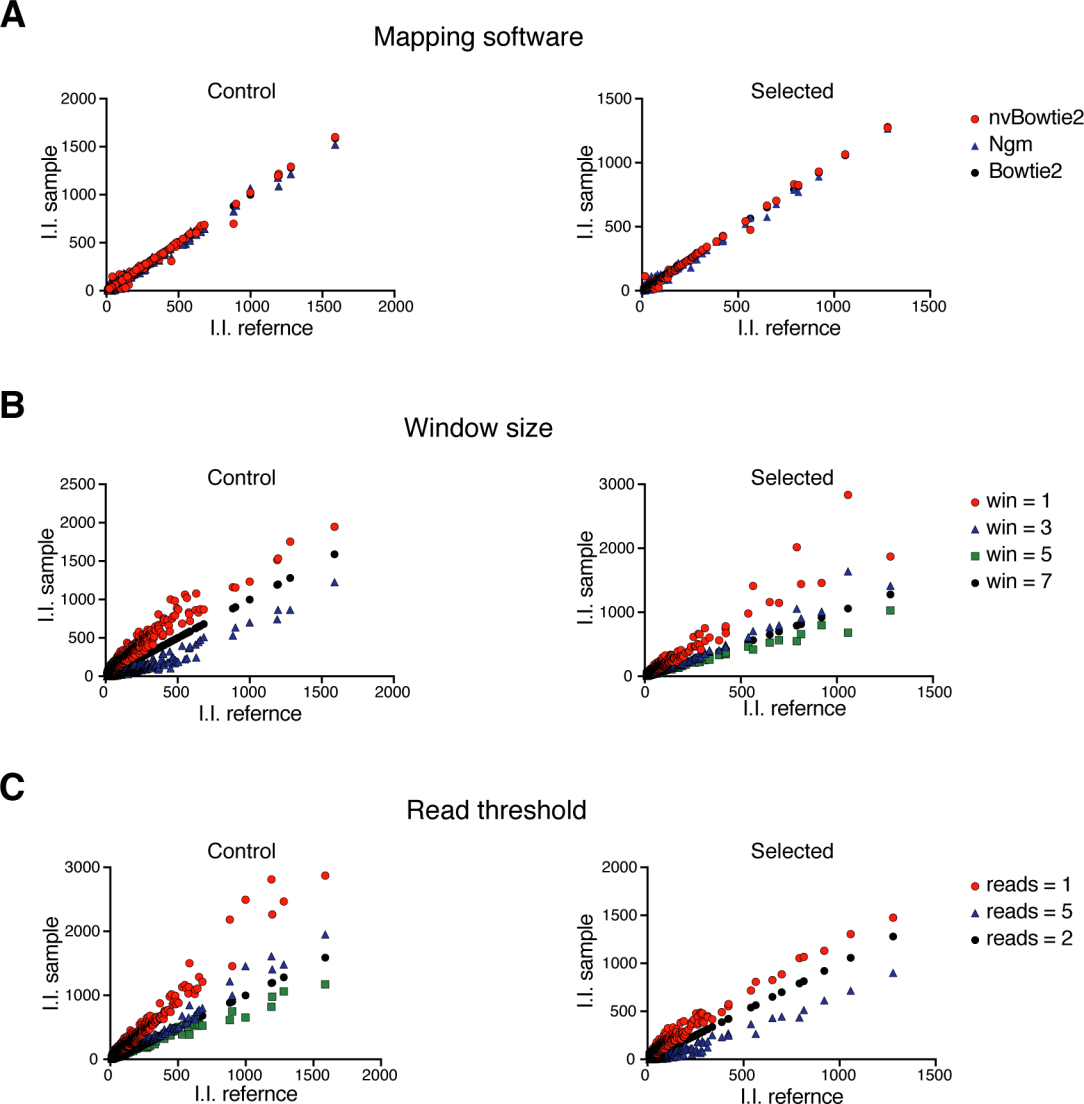
Effects of analysis parameters on candidate identification. (**A**) Comparison of the number of I.I. that were obtained by using different read mapping tools. Genes with more than 10 insertions are plotted and the sample aligned with Bowtie2 is used as reference (I.I. reference). (**B**) Effect of genomic window size on defining I.I. from reads. Genes with more than 10 insertions are plotted and a 5 nucleotide window is used as reference (I.I. reference). (**C**) Effect of read number threshold on defining I.I.. Genes with more than 10 insertions are plotted and a threshold for 2 reads is used as reference (I.I. reference).

We developed HaSAPPy by analysing a screen for silencing factors in X inactivation [18], which used an inducible *Xist* expression system in haploid mouse ESCs for identifying mutations that mediate cell survival by escaping inactivation of the X chromosome. 7 control (SRX1060416) and 7 selected (SRX1060407) datasets with a total of 300 million reads were analysed on workstations with Ubuntu Linux version 14.04, at least 32 gigabytes memory, and graphics processing units (GPUs). Runtimes between 90 minutes and 3 hours were predominated by read mapping and subsequent analyses were also performed on a Macbook (Intel Core i7, 2.3GHz) in 30 minutes. We have evaluated different read mappers and alignment parameters by benchmarking on experimental and generated read datasets using a previously published test suite [26]. As a result HaSAPPy is preconfigured to run with Bowtie2 [24], nvBowtie, and NextGenMap [25]. The latter two aligners utilize accelerators for speeding up read alignment by taking advantage of recent hardware developments. Although some differences in the number of insertions assigned to candidate genes were observed (Fig 3A, S2 Fig, Table A in S1 Text), our results suggest that GPUs can be effective in speeding up read mapping without a loss in sensitivity consistent with earlier results [25,26].

### Reconstruction of independent virus insertion events

**Fig 4:**
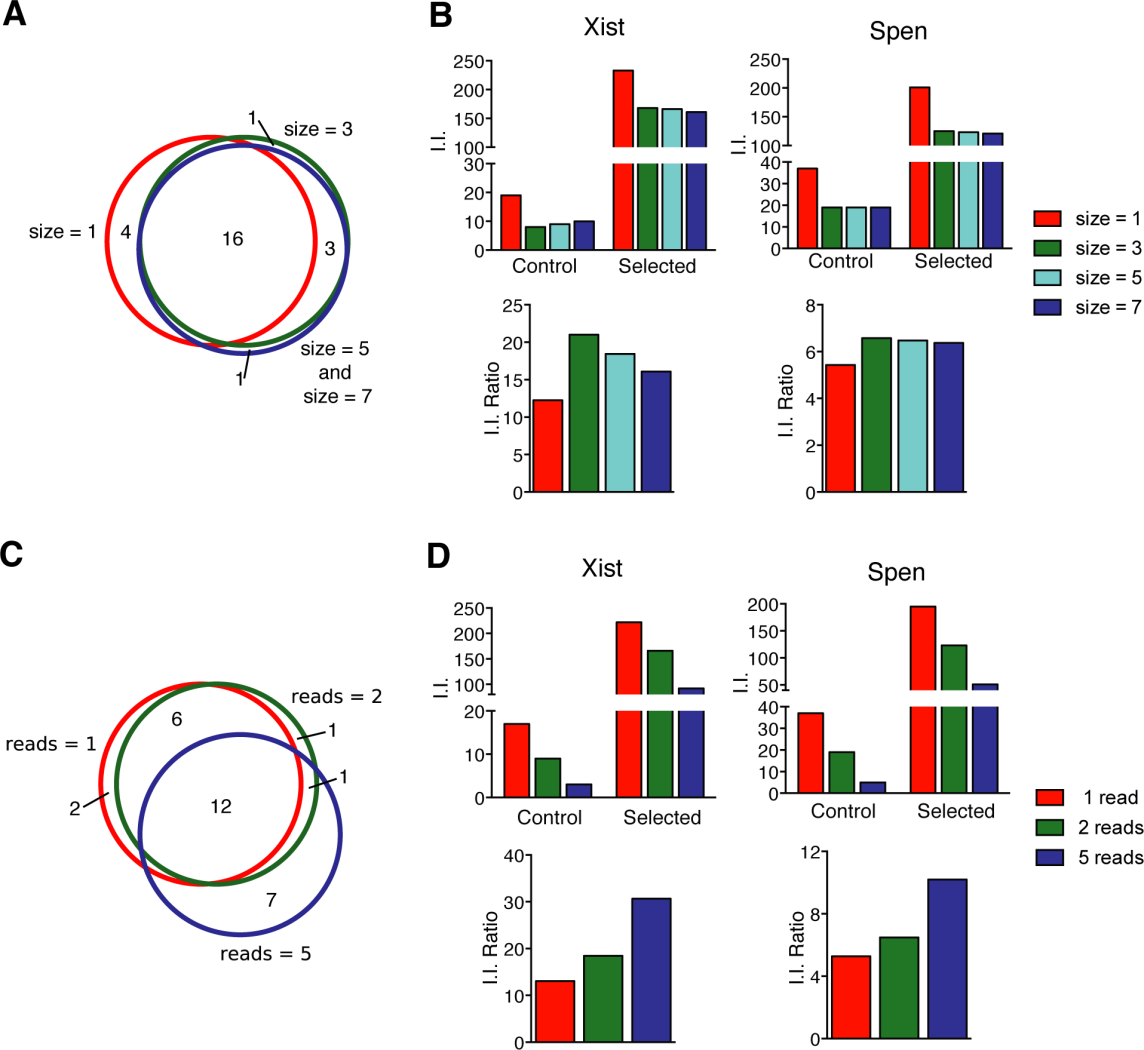
Effects of analysis parameters on candidate ranking. (**A-B**) Effects of variation in the window size applied to define I.I. in candidate selection. (**A**) Venn-diagram comparing the 20 top candidates. (**B**) Effect of window size on I.I. for the experimentally validated candidates *Xist* and *Spen*. The number of I.I. and the ratios of I.I. in Selected over Control samples for different window sizes are shown. (**C-D**) Effects of the read number threshold for I.I. on candidate identification. (**C**) Venn-diagram comparing the 20 top candidates. (**D**) Insertion number (I.I.) of *Xist* and *Spen* using different thresholds.

Independent insertions are reconstructed from read alignments for estimating the selection of mutations in potential candidate genes over background. Typically, each insertion results in multiple reads, whereby read counts reflect amplification and library preparation as well as the abundance of insertions in the cell populations [22]. We find that individual insertions can be represented by more than 10^5^ reads. Their mapping to isolated genomic regions without annotated genes hints at strong amplification bias. To ensure that only insertions that have been selected independently are scored, we consider for each dataset at most one insertion for each genomic position by combining all reads starting within a genomic window on the same strand into a single independent insertion (I.I.). The genomic position with the highest initial read count is reported along with the cumulative read count (Fig 1C). We evaluated the effects of the window size on candidate ranking (Fig 3B, Fig 4A and 4B, and Table B in S1 Text). Although, increasing window sizes did not affect the ranking of the highest scoring genes, variation was observed for lower ranked candidates. We determined that a window size larger or equal to 5 nucleotides resulted in greater reproducibility. The average distance between independent viral insertions can be expected to be larger than 5 bases suggesting that loss of information is negligible. Trimming or sequence errors cause alignment shifts of few nucleotides and are corrected by our approach.

Read alignment errors can affect the analysis of genetic screens, where a single read can be sufficient for the reconstruction of a viral insertion event. To mitigate this problem, we introduce a threshold for the number of reads that is required for considering an insertion in the analysis. Selecting stringent thresholds reduces the number of I.I., which is particularly problematic for unselected libraries with large numbers of insertions and lower read coverage (Table C in S1 Text). Also candidate genes that are supported by insertions with low read coverage become undetectable (Fig 3C, Fig 4C and 4D) reflecting a reduction in sensitivity. A threshold of 2 reads to consider an insertion can reduce noise from alignment errors without materially reducing I.I. numbers. Additionally, different read number thresholds can be used for adjusting for sampling rates in control and selected datasets.

### Comparison of candidate ranking strategies

**Fig 5:**
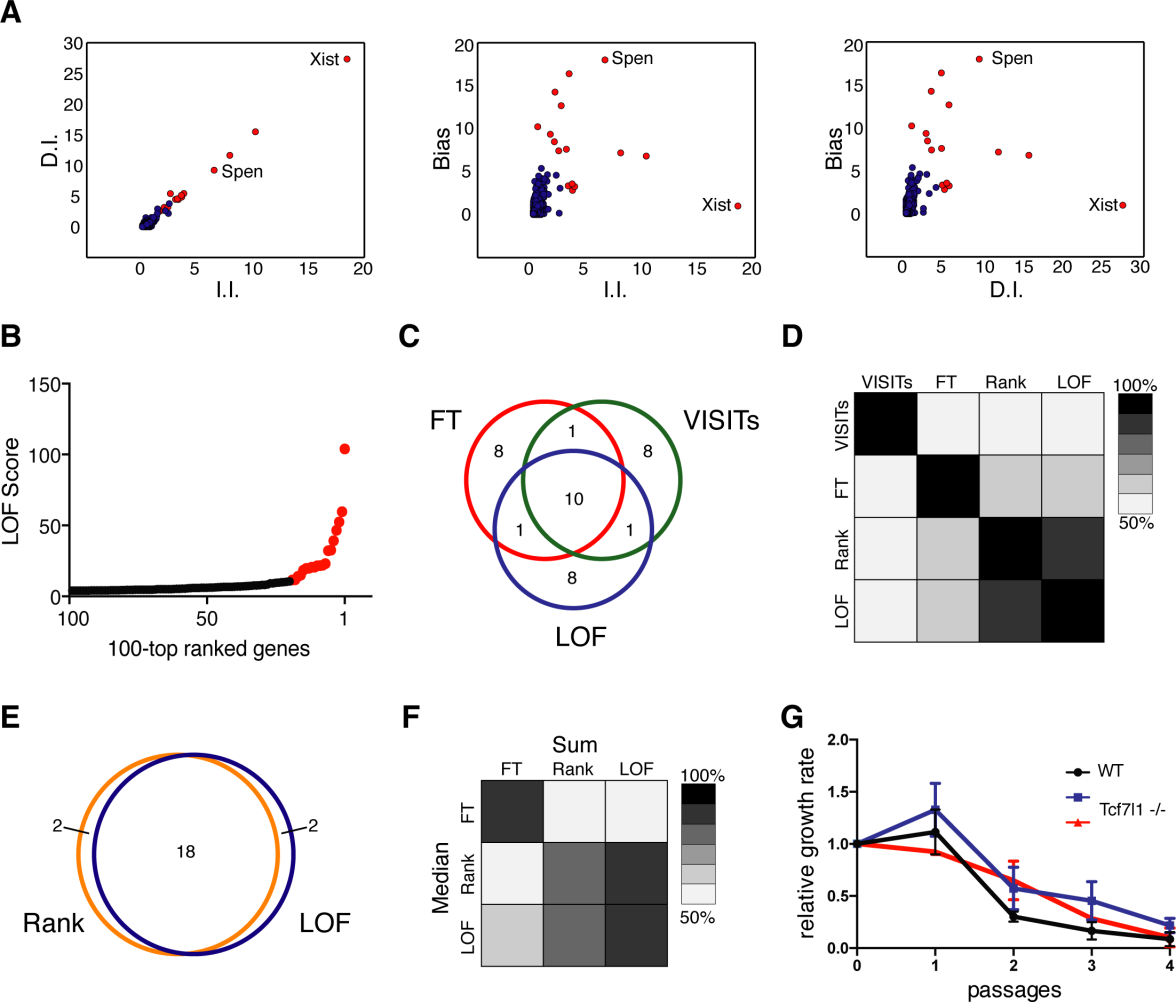
Comparison of candidate selection algorithms. (**A**) Visualization of fold enrichment of I.I. and D.I. (left panel), I.I. and Bias (Central panel), and D.I. and Bias (right panel) during selection. The 20 top ranked genes by the LOF algorithm are shown in red, and the validated candidates *Xist* and *Spen* are annotated. (**B**) LOF score of the first 100 genes of the candidate list plotted against position. The 20 top ranked genes are shown in red. (**C**) Venn-diagram showing overlap of the 20 top ranked candidates identified by FT, VISITs, and LOF. (**D**) Heatmap showing correlation between candidate lists of FT, VISITs, Rank and LOF. 20 top candidates were considered, colour coding indicates the percentage of genes that are in common. (**E**) Venn-diagram showing 20 top candidates using Rank and LOF algorithms. (**F**) Heatmap showing overlap of the 20 top candidates by FT, Rank, and LOF algorithms using either the entire dataset (Sum) or the median of 7 replicates (Median). Colour coding as in panel D. (**G**) Relative growth rate of 2 *Tcf7l1* deficient ESC clones (red and blue) and WT control ESCs (black) after *Xist* induction with Doxycycline is plotted. Error bars represent standard deviation (n = 3).

For obtaining a candidate list a scoring function or selection strategy needs to be implemented. Previous methods have applied Fisher's exact test (FT) for detecting enrichment of D.I. in selected compared to control samples [20]. VISITs implements a statistical candidate selection strategy by combining FT for D.I. enrichment and a binomial test for comparing D.I. to I.I. in selected samples [22]. Candidates are subsequently ordered by increasing probability of no difference between selected and control datasets. FT based methods have not considered quantification of additional biological parameters for evidence of selection. Rank-based methods can also be applied for sorting candidates, whereby genes are assigned a rank according to their number of D.I., and the difference of the logarithmic rank positions of a gene in the selected and control datasets is used for candidate ranking by sorting for increasing values. Although, these strategies are generally effective they do not take full advantage of gene structure information. To improve on this situation, we developed a new algorithm based on two considerations. Firstly, multiple parameters are evaluated for each gene in parallel for detecting evidence for selection. Secondly, evaluation of parameters is performed relative to all other genes. For each gene, we construct a vector using the fold enrichment of I.I., D.I. and Bias values between selected and control samples. The distance from the mass centre of all genes in 3-dimensional vector space is used to obtain a score by the Local Outlier Factor (LOF) algorithm [28]. Candidates are identified and scored as most diverging genes similar to outliers (Fig 5A and 5B, S3A Fig and Table 1).

**Table 1:** LOF selected candidates.

Gene	Score	II	DI	Bias
Xist	104.0	18.4	27.3	1.0
Hira	59.7	10.2	15.5	6.8
Spen	52.5	6.5	9.2	18.0
Med25	46.6	8.1	12.0	7.4
Cdk8	39.2	3.6	5.0	18.4
Kdm5b	32.6	2.0	3.2	14.3
Tcf7l1	32.2	2.5	5.4	12.7
Eed	23.0	3.0	4.5	7.6
Cat	21.9	0.6	1.0	14.8
Fbrsl1	21.7	1.6	2.5	9.4
Rbm12	21.5	4.4	6.3	3.8
Fbxw7	20.6	1.9	2.7	8.5
Ubn2	20.6	6.4	9.3	6.5
Nsd1	19.8	2.3	3.2	7.4
Cpne1	19.6	4.1	5.6	3.3
Smarcad1	18.5	3.2	4.6	3.3
Scaf8	15.0	2.5	3.9	3.2
Suz12	14.1	2.1	2.7	4.7
Specc1l	11.7	0.7	0.8	5.4

We compared the performance of different candidate ranking strategies by applying methods available in HaSAPPy (FT, Rank, LOF) and VISITs [22] for analysis of the X inactivation datasets aligned with Bowtie2 (Fig 5C and Table D in S1 Text). Ten genes were shared among the first twenty predicted candidates by all methods, whereby differences were observed in the ranking order (S4 Fig). The LOF and Rank methods showed substantial overlap (Fig 5D and 5E) despite the different algorithms and parameters. Candidates predicted by VISITs and FT showed limited overlap, which is likely explained by implementation of additional data transformations in VISITs. We next evaluated candidates by examining the orientation of insertions in introns (Bias [29]) as evidence for selection. We observed an overall lower Bias for genes that were uniquely predicted by FT and VISITs than by LOF (Table E in S1 Text). To further evaluate the robustness of candidate prediction, we segregated the 7 replicates of the X inactivation dataset and treated them as independent experiments. Candidate lists obtained by using the median number of insertions in control and selected samples substantially overlapped with lists obtained from using the entire dataset and so did their relative order (Fig 5F and Table F in S1 Text). Lower ranked candidates with few insertions were lost in the lists of median Rank and LOF analysis reflecting the subsampling of the data, whereas candidates specifically predicted by FT showed substantial divergence. In conclusion, replicates can increase the robustness of analyses, but the overall number of I.I. is correlated with sensitivity for detecting potential candidates.

*Xist* and *Spen* were experimentally validated [18], and ranked top and third in the lists of the Rank and LOF methods (Table D in S1 Text). FT or VISITs produce large selections of candidates in which *Xist* and *Spen* are present. Effectively, these occupy lower positions. *Tcf71l* is predicted as a potential candidate by all methods and suggested at the top of the list output by VISITs and FT when sorted for decreasing probability of no difference. For this reason we were interested in evaluating *Tcf7l1* experimentally and engineered a mutation in the first exon using CRISPR/Cas9 nucleases. Loss of Tcf7l1 protein was confirmed by Western analysis (S8 Fig). In HATX3 ES cells, a Doxycycline inducible *Xist* allele facilitates to study the effect of a *Tcf7l1* mutation on *Xist* function [18]. Induction of *Xist* caused a similar cell loss in the parental and *Tcf7l1* deficient HATX3 ESCs (Fig 5G), which was not comparable to increased survival caused by a mutation in *Spen* [18]. Therefore, *Tcf7l1* is likely not involved in *Xist* mediated gene repression. Selection of *Tcf7l1* might be explained by a generally enhanced self-renewal and reduced differentiation of *Tcf7l1* deficient mouse ESCs [30]. Hence, what appears to be detected is a positive selection for *Tcf7l1* mutations in mouse ESCs, which is unrelated to a loss of *Xist* function. For elimination of such candidates additional considerations are required.

### Visualization of insertions for candidate validation

We implemented a graphical view of insertions in candidate gene loci into HaSAPPy that facilitates a comparison between selected and control samples. Colour coding is used for sense and anti-sense insertions for visualization of a selection for disruptive over neutral mutations within introns. We observe an increase of insertion numbers in selected samples for genes that were identified by all ranking strategies (FT, VISITs, Rank and LOF) and the enrichment for mutagenic insertions can be confirmed (S4 Fig). Additionally, genes that are uniquely predicted by the LOF algorithm have similar properties (S5 Fig). In contrast, the candidate list produced by the FT method (S6 Fig) contains genes characterized by a high number of mutations in both selected and control samples. This peculiarity was also observed in VISITs (S7 Fig) suggesting that insertion number independently from evidence of selection influences the prediction of candidates by FT based methods.

### Benchmarking candidate prediction

**Fig 6:**
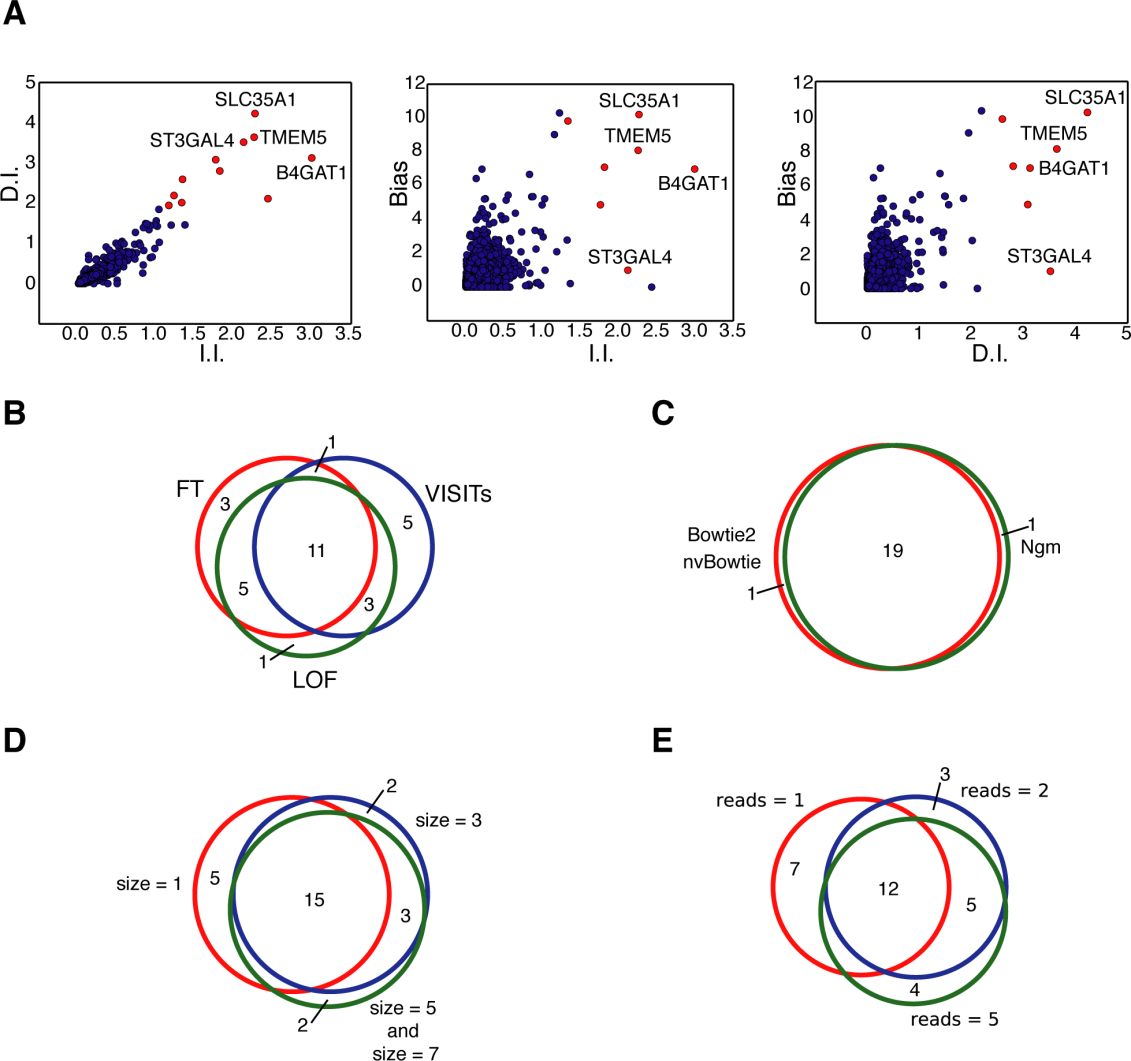
Benchmarking HaSAPPy on a screen for resistance to Lassa virus entry. (**A**) Visualization of parameter distribution for the LOF algorithm. For each gene the fold enrichment after selection in I.I. and D.I. (left panel), I.I. and Bias (Central panel) and D.I. and Bias (right panel) is plotted. The 12 top ranked genes by the LOF are marked in red, and validated candidates are annotated. (**B**) Venn-diagram comparing 20 top candidates predicted by the FT, VISITs and LOF algorithms. (**C**) Venn-diagram showing overlap of the 20 top ranked candidates using Bowtie2, nvBowtie and NextGenMap (Ngm) for read alignment. (**D**) The Venn-diagram shows the overlap of the 20 top candidates using different window sizes, and (**E**) using different read number thresholds for including an I.I. in the analysis.

For comprehensively benchmarking HaSAPPy we reanalysed several published screens in human haploid cells for resistance to virus entry [8,20]. These studies differ from the screen of silencing factors in X inactivation in two important ways. Firstly, the screens were performed in human haploid cells and therefore require the use of the human genome and annotation for the analysis. Secondly, the datasets were generated using an Illumina HiSeq instrument and consist of much higher read numbers of 30 nucleotide length, which is shorter than the read length in our *Xist* screen [18]. HaSAPPy was run using our previously determined parameters. The top 4 predicted candidates by the LOF algorithm have been experimentally validated factors for Lassa virus entry [20] suggesting that HaSAPPy detects candidates accurately and with similar success as the manually curated strategy of the original study (Fig 6A and 6B, S9 Fig and Table G in S1 Text). Similar results were obtained for the datasets of Staring et al. [8]. PLA2G16 has been validated to be essential for Poliovirus, Coxsackievirus B1 and Coxsackievirus A7 infections and was predicted as top candidate by HaSAPPy (S10 Fig and Table K in S1 Text). In addition, we find evidence for selection of NBPF20 through a high enrichment in D.I. mutations. NBPF20 has not been detected in the original study. Taken together, the performance of HaSAPPy with preset parameters in a total of 5 different screens [8,18,20] suggests a wide range of applications over different genomes and sequencing technologies. The robustness against changes in data analysis parameters (Fig 6C–6E and Table H–J in S1 Text) facilitates the adaptation and use of HaSAPPy as a standardized analysis method for haploid cell genetic screens.

## Discussion

Analyses of genetic screens require sensitivity to avoid missing individual candidates. Pathways can be represented by single candidates, when redundancy or lethality reduces opportunities of discovery. Conversely, false predictions can lead to costly experimental validation, which limits the number of genes that are followed up. Therefore there is a need for effective candidate ranking. NGS datasets from genetic screens require strategies to identify evidence for selection and reduce technical noise that differ from other sequencing analysis problems. Our study introduces three methodical procedures for candidate prediction in haploid screens.

Firstly, we aim to accurately reconstruct viral insertion events from read alignments while eliminating effects from amplification bias, sequencing and alignment errors. In HaSAPPy, reads within a genomic window are used to reconstruct a viral insertion event that is guaranteed to have been selected independently. This procedure is unlikely to remove truly independent insertions, but will remove effects from alignment shifts caused by sequencing and amplification errors. Window size and threshold for the number of reads for defining an insertion can be adjusted to further increase the robustness of candidate selection.

Secondly, HaSAPPy scores multiple parameters for each gene simultaneously to detect evidence for selection. Considering the mutagenic effect of intronic insertions ensures that candidates with a wide range of possible gene structures can be detected. Whereas an increase in the total number of insertions can be expected for selected genes, statistics can be less sensitive for small genes. Multiple viral integrations per cell can lead to co-selection of a driver mutation with passenger mutations that are not causal to the phenotype. Genes that are in genomic regions with frequent virus insertions can maintain high numbers of insertions through co-selection. Additional evidence for selection can come from examining disruptive mutations, and the ratio between disruptive and neutral intronic mutations can be useful to detect evidence of selection if large introns are present. Scoring these three parameters simultaneously in a mathematically consistent framework is achieved by using a multidimensional outlier method. We verified that this approach is effective in predicting candidates from 5 different screens. HaSAPPy is able to rank candidates in order of decreasing evidence for selection and performs equal or better than previously used methods. Co-selection of passenger mutations may affect the predictions of FT based algorithms leading to a larger set of candidates that are not sorted for biological evidence for selection. In addition, HaSAPPy compensates experiment specific effects that act on all genes using an outlier detection strategy without a need for normalizing datasets. Complex library preparation, amplification and insertion biases in haploid screening can lead to a lack of homogeneity in insertion coverage between samples. Normalization strategies are therefore difficult to implement and risk distorting the experimental information through unanticipated effects of data transformation.

Finally, for enabling researchers to add biological expertise and literature to candidate evaluation, HaSAPPy makes all parameters of the analysis accessible in a customizable table format. A graphical overview of insertions in candidate genes supports the user with selecting candidates for further studies.

### Availability and future directions

HaSAPPy is implemented in Python and released as open-source software under the MIT license. It supports many typical haploid screening projects and can be adapted to experimental designs and candidate ranking strategies. Presently, HaSAPPy does not utilize transcript datasets of haploid cells [31]. In future versions candidates for which little evidence of transcription is observed could be eliminated and gene models could be refined. Development of graphical user interfaces for specifying run parameters could further facilitate interactive use of HaSAPPy on workstations. It will also be enticing to explore methods for improving read alignment of screening datasets, which often contain short reads and sequence errors. A preliminary analysis indicates that nearly half of all viral insertions occur within genes.

### Datasets, genome assemblies, and accession numbers

Source code is available from https://github.com/gdiminin/HaSAPPy. Datasets of the X inactivation screen can be downloaded from the SRA archive (https://www.ncbi.nlm.nih.gov/sra) and consists of 7 control (SRX1060416) and 7 selected samples (SRX1060407). The Lassa Virus resistance dataset consists of 1 control (SRR663777) and 1 selected (SRR656615) sample. The Picornavirus resistance dataset consists of 1 control (SRR663777) and 3 selected (PV1-SRR4885982, CVB1- SRR4886610, CVA7- SRR4887274) samples. Alignments to the mouse and human genomes were performed using the UCSC mm10 and USCS hg38 assemblies, respectively.

## Supporting information

S1 Text Supporting information. (DOCX)

S1 Fig Screen capture illustrating HaSAPPy output. (TIF)

S2 Fig Effects of read mapper choice on candidate ranking. (**A-B**) Venn-diagram comparing the 20 highest ranked candidates using Bowtie2, nvBowtie and NextGenMap for read alignment. Candidate predictions were performed using the LOF algorithm on fold enrichment (**A)** and rank (**B**) datasets. (**C**) Number of I.I. in *Xist* and *Spen* using different read aligners in control and selected samples and the ratio thereof.
(TIF)

S3 Fig HaSAPPy candidates in X inactivation screen (Monfort et al., 2015). (**A**) Plot of genes represented according to fold enrichment during selection in I.I., D.I. and Bias. The 20 top ranked genes using the LOF algorithm are shown in red. The positions of *Xist* and *Spen* are annotated. (**B**) Distribution of I.I. at the level of genes detected by HaSAPPy and biologically validated in Monfort et al., 2015. Selected (top panel) and Control (bottom panel) samples are compared. Insertions occurring in the orientation of gene transcription are marked in red, anti-sense insertions are marked in green.
(TIF)

S4 Fig Candidates identified by all ranking strategies. Distribution of I.I. at the level of genes detected by the different algorithms. Selected (top panel) and Control (bottom panel) samples are compared. Insertions occurring in the orientation of gene transcription are marked in red, anti-sense insertions are marked in green.
(TIF)

S5 Fig Candidates identified specifically by the LOF analysis. Distribution of I.I. within genes detected by the LOF algorithm (Outlier-only). Selected (top panel) and Control (bottom panel) samples are compared. Insertions occurring in sense and antisense orientation of gene transcription are marked in red, and green, respectively.
(TIF)

S6 Fig Candidates identified specifically by the Fisher analysis. Distribution of I.I. within genes detected by the FT algorithm (Fisher-only). Selected (top panel) and Control (bottom panel) samples are compared. Insertions occurring in sense and antisense orientation of gene transcription are marked in red, and green, respectively.
(TIF)

S7 Fig Candidates identified specifically by VISITs analysis. Distribution of I.I. within genes detected by VISITs algorithm (VISITs-only). Selected (top panel) and Control (bottom panel) samples are compared. Insertions occurring in sense and antisense orientation of gene transcription are marked in red, and green, respectively.
(TIF)

S8 Fig Generation of Tcf7l1 deficient ESCs lines. Western analysis of two *Tcf7l1* deficient (-/-) and one control (WT) HATX3 ES cell clones. Commassie Blue staining is used to control for loading (below).
(TIF)

S9 Fig HaSAPPy candidates in the human haploid screening for factors involved in resistance to Lassa Virus entry (Jae et al., 2013). **A**) Plot of genes represented according to fold enrichment during selection in I.I., D.I. and Bias. The 12 top ranked genes using the LOF algorithm are shown in red. The positions of *SLC35A1*, *B4GAT1*, *TMEM5*, and *ST3GAL4* are annotated. (**B**) Distribution of I.I. at the level of genes detected by HaSAPPy and biologically validated in Jae et al., 2013. Selected (top panel) and Control (bottom panel) samples are compared. Insertions occurring in the orientation of gene transcription are marked in red, anti-sense insertions are marked in green.
(TIF)

S10 Fig HaSAPPy candidates in the human haploid screening for factors involved in Picornaviridae Virus entry (Staring et al., 2017). (**A**) Plot of genes represented according to fold enrichment during selection in I.I., D.I. and Bias for Poliovirus (PV1), Coxsackievirus B1 (CVB1) and Coxsackievirus A7 (CVA7) infection. Genes characterized by a LOF value higher than 15 are shown in red. Genes with a LOF value higher than 20 are annotated in the plot. (**B**) Distribution of I.I. within genes detected by the LOF algorithm and in common among the different selection strategies. *PLA2G16* was biologically validated in Staring et al., 2017. Selected (PV1, CVB1 and CVA7—top panels) and Control (bottom panel) samples are compared. Insertions occurring in sense and antisense orientation of gene transcription are marked in red, and green, respectively.
(TIF)
