# Supplementary material for: HaSAPPy: A tool for candidate identification in pooled forward genetic screens of haploid mammalian cells
Source: PLoS Comput Biol. 2018 Jan 16;14(1):e1005950. doi: 10.1371/journal.pcbi.1005950 (PMC5798846; doi:10.1371/journal.pcbi.1005950)
Supplement: S1 Text — (DOCX) [file pcbi.1005950.s001.docx]

**Supplementary Information**

Supplementary Materials

**Cell culture and treatment**

Derivation of haploid ESCs from *Xist*TX/TX R26nlsrtTA/nlsrtTA mice was previously described [18]. HATX3 ES cells were grown in 2i (containing PD0325901, CHIR99021, and LIF) medium. Doxycyclin (1ug/ml) was used to induce *Xist* expression in survival assay.

**Generation of Transgenic Cell Lines**

Tcf7l1 loss of function ES cells were generated using a single gRNA CRISPR/cas9 strategy. The gRNA targeting the first exon (gRNA: CGGCCGAGCAGCGACAGCGCTT) was inserted into the pX458-pSPCas9(BB)-2A-GFP vector (Addgene, # 48138). Briefly, the vector was lipofected into HATX3 cells and sorted for GFP fluorescence after 48hrs. Clonal cell lines were obtained by low density plating and loss of Tcf7l1 protein was confirmed by immunoblot using goat α-Tcf7l1 antibody (M20) (SCBT, SC-8635)

Supplementary Tables

Table A: Alignment software

Table B: Window size to define I.I.

Table C: Cut-off to define I.I.

Table D: Algorithms for candidate selection

Table E: Bias values of genes uniquely identified by ranking algorithms

Table F: Candidate ranking using median values

Table G: Algorithms for candidate selection in haploid screening for resistance to Lassa Virus entry (Jae et al. 2013)

Jae et al.

Comparison of the top 20 candidates obtained using different algorithms for candidate selection (LOF, Original list, FT and VISITs) . In the original paper (Jae et al.) candidates were selected using the Fisher's exact test (FT) for statistical evaluation of Disruptive Insertions. Genes with a percentage of intronic region higher than 70% were further evaluated for the enrichment of intronic sense insertions using a binomial test (Binomial Test). Remaining genes were re-evaluated with FT applying more restrictive criteria. The genes functionally validated in the original work are highlighted in yellow.

Table H: Alignment software in Jae et al. 2013 database

Table I: Window size to define I.I. in Jae et al. 2013 database

Table J: Cut-off to define I.I. in Jae et al. 2013 database

Table K: Candidate selection in haploid screening for resistance to Picornaviridae Virus entry (Staring et al. 2017)

Comparison of the top candidates obtained using different algorithms for candidate selection (LOF, Original list). In the original paper (Staring et al.) candidates were selected using the Fisher's exact test (FT) for statistical evaluation of Disruptive Insertions. The gene functionally validated in the original work is highlighted in yellow.
