# Supplementary figures and images for: HaSAPPy: A tool for candidate identification in pooled forward genetic screens of haploid mammalian cells
Source: PLoS Comput Biol. 2018 Jan 16;14(1):e1005950. doi: 10.1371/journal.pcbi.1005950 (PMC5798846; doi:10.1371/journal.pcbi.1005950)

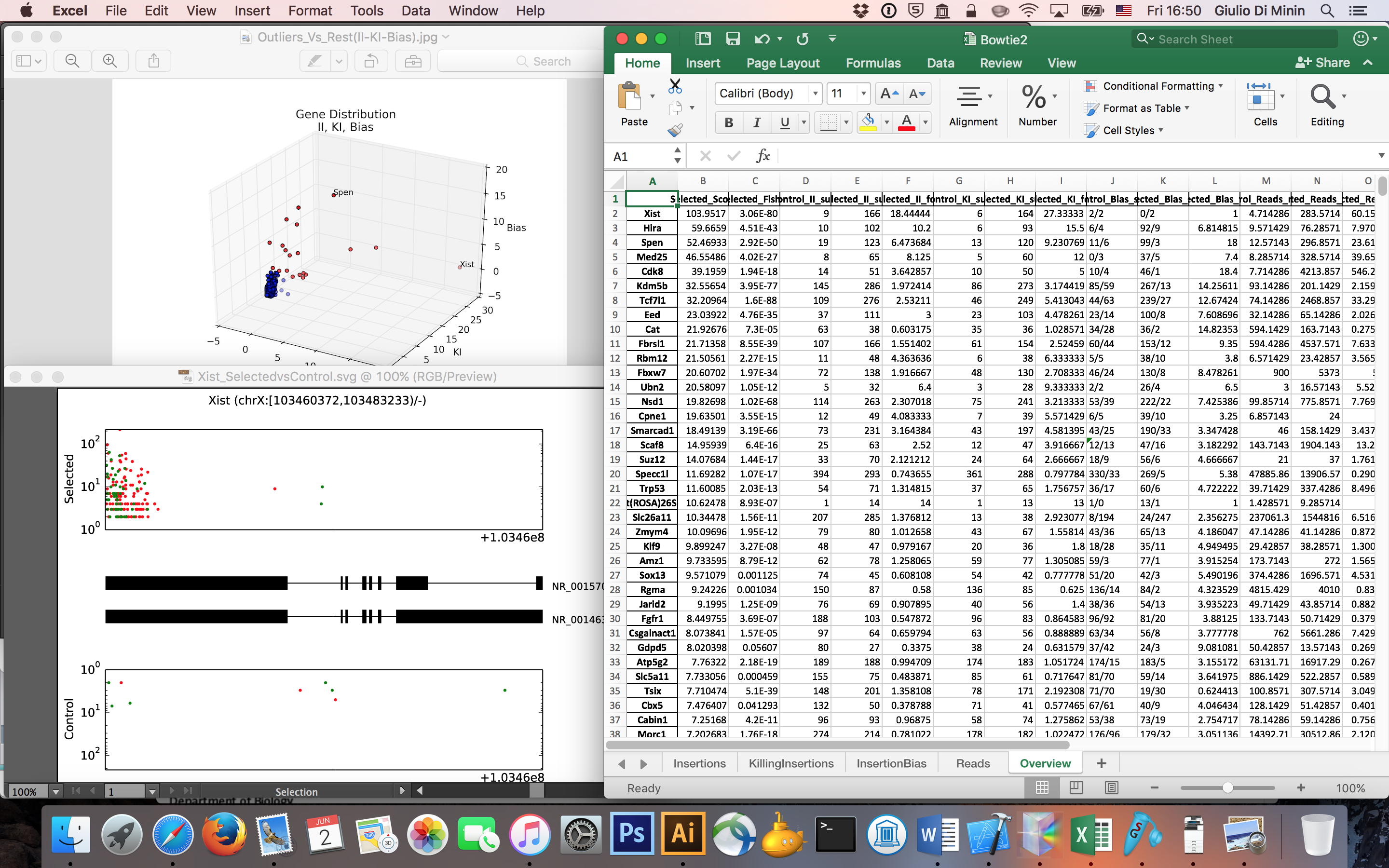

Supplement: S1 Fig — (TIF) [file pcbi.1005950.s002.tif]

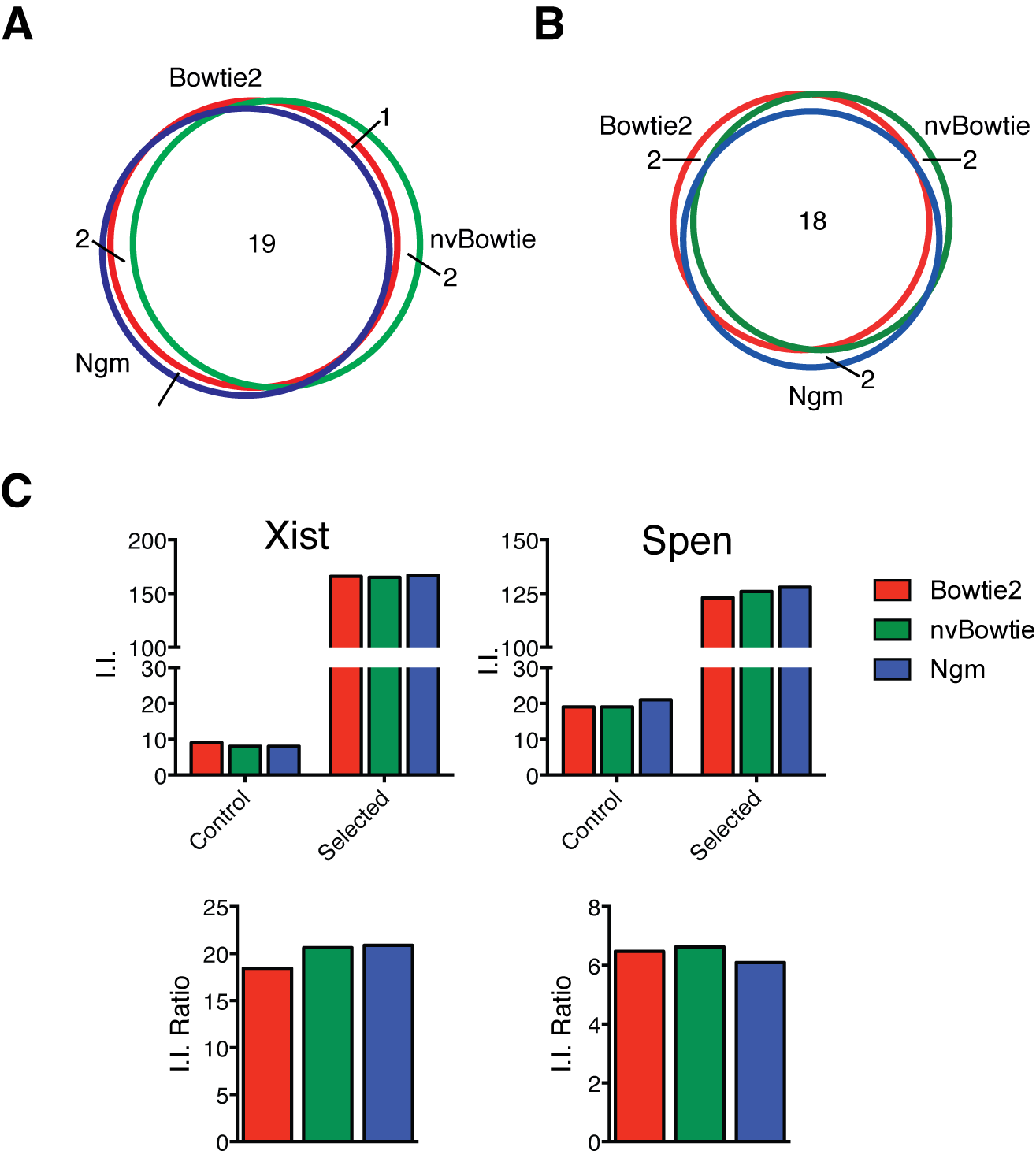

Supplement: S2 Fig — (A-B) Venn-diagram comparing the 20 highest ranked candidates using Bowtie2, nvBowtie and NextGenMap for read alignment. Candidate predictions were performed using the LOF algorithm on fold enrichment (A) and rank (B) datasets. (C) Number of I.I. in Xist and Spen using different read aligners in control and selected samples and the ratio thereof. (TIF) [file pcbi.1005950.s003.tif]

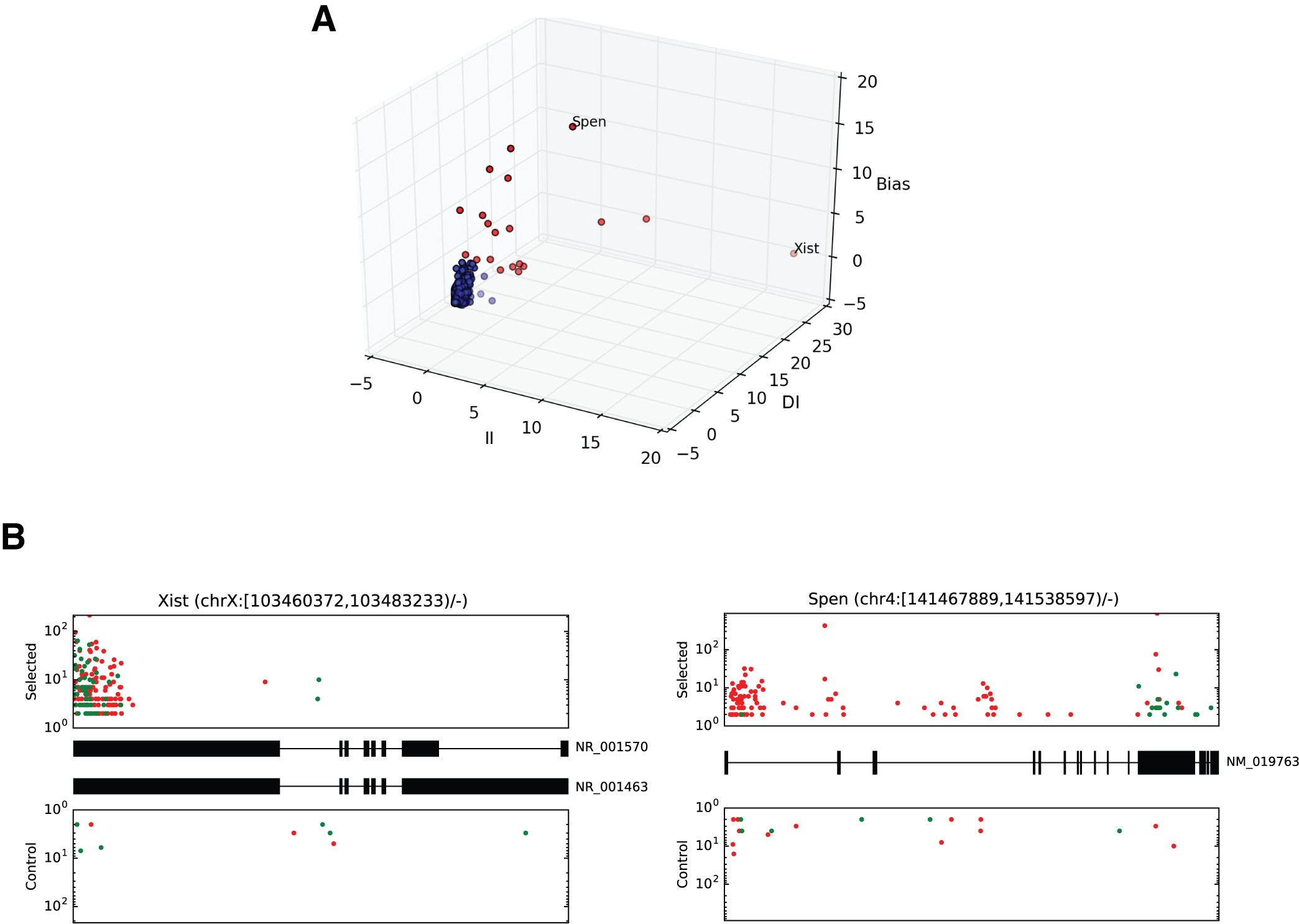

Supplement: S3 Fig — (A) Plot of genes represented according to fold enrichment during selection in I.I., D.I. and Bias. The 20 top ranked genes using the LOF algorithm are shown in red. The positions of Xist and Spen are annotated. (B) Distribution of I.I. at the level of genes detected by HaSAPPy and biologically validated in Monfort et al., 2015. Selected (top panel) and Control (bottom panel) samples are compared. Insertions occurring in the orientation of gene transcription are marked in red, anti-sense insertions are marked in green. (TIF) [file pcbi.1005950.s004.tif]

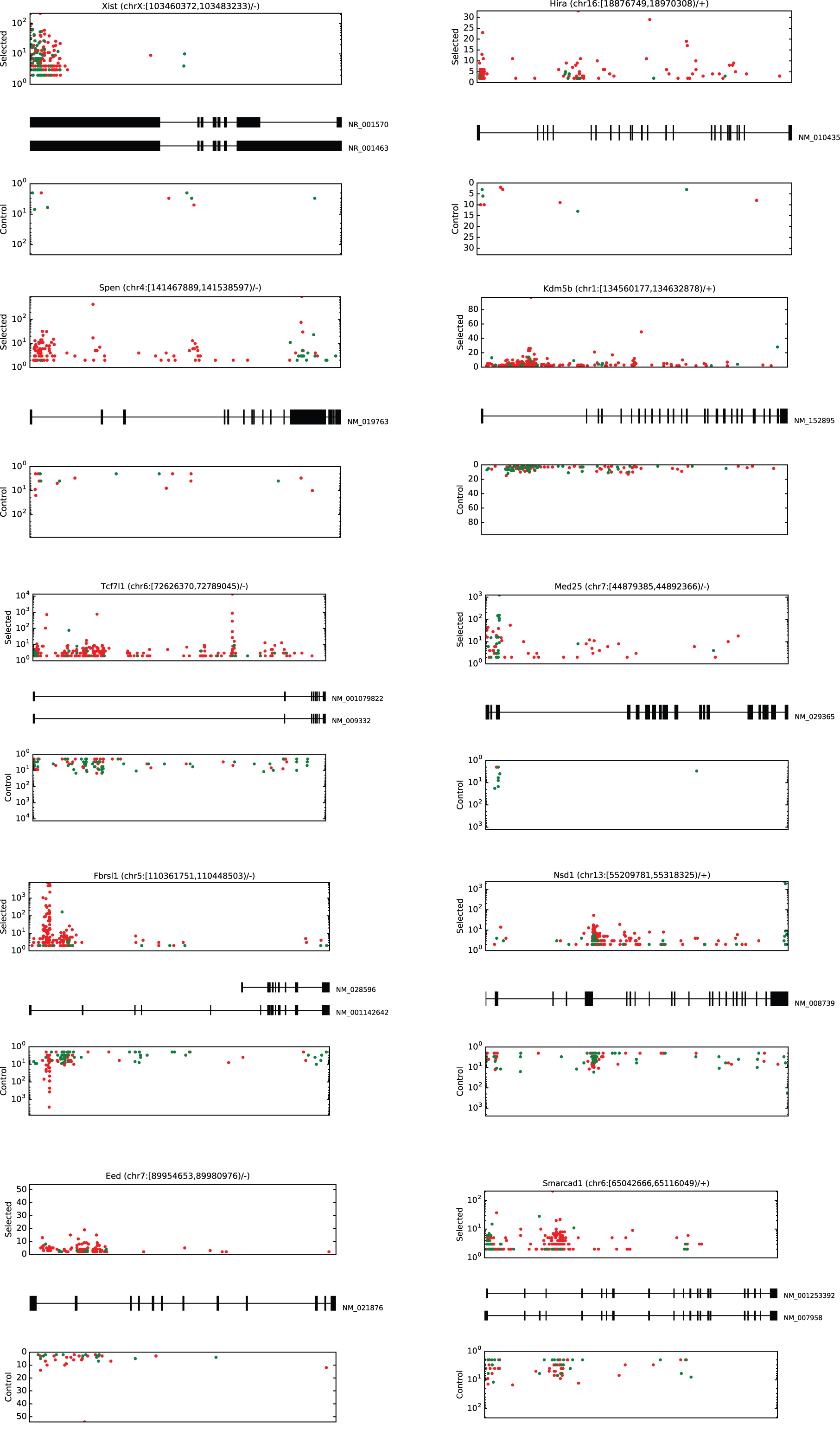

Supplement: S4 Fig — Distribution of I.I. at the level of genes detected by the different algorithms. Selected (top panel) and Control (bottom panel) samples are compared. Insertions occurring in the orientation of gene transcription are marked in red, anti-sense insertions are marked in green. (TIF) [file pcbi.1005950.s005.tif]

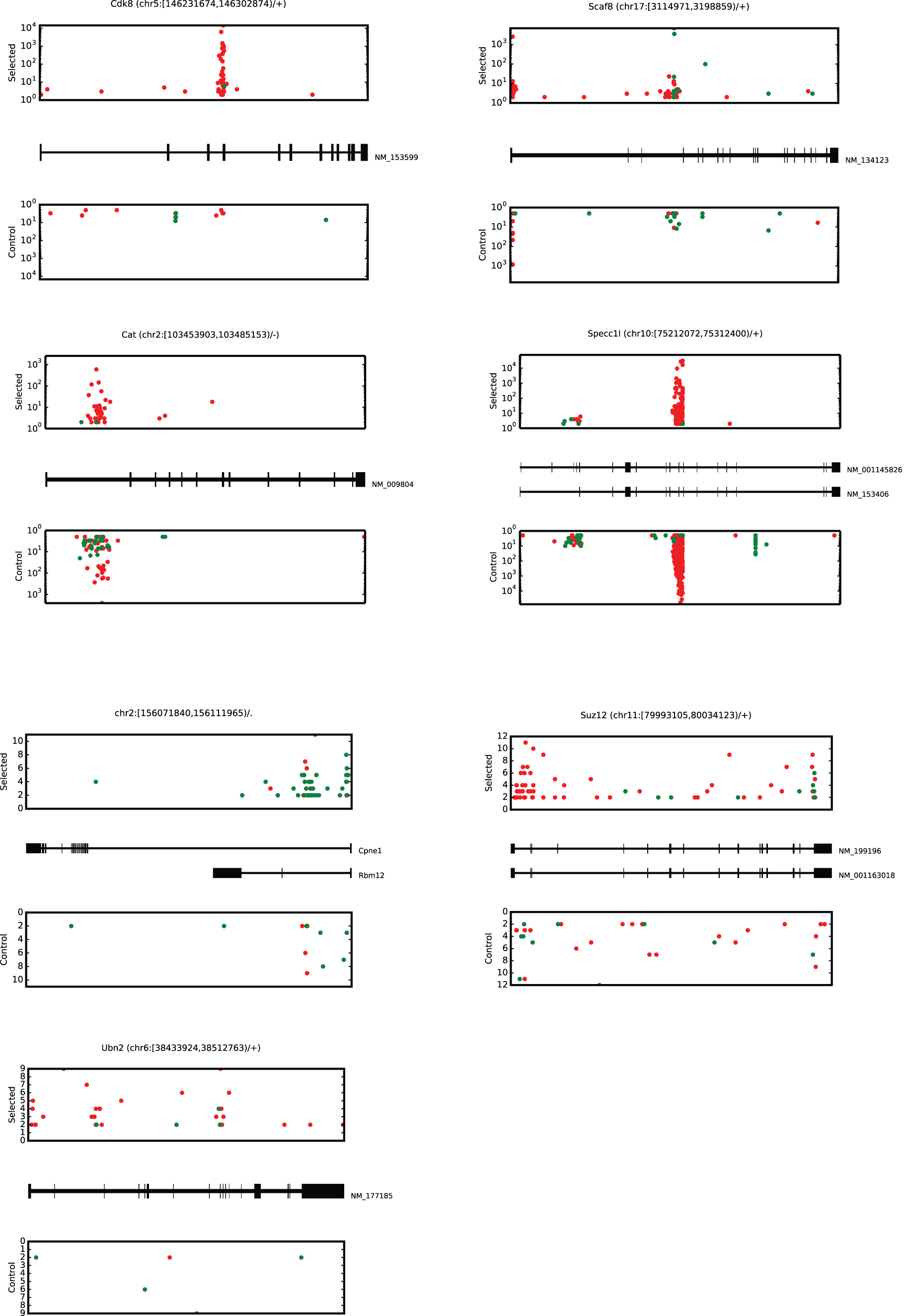

Supplement: S5 Fig — Distribution of I.I. within genes detected by the LOF algorithm (Outlier-only). Selected (top panel) and Control (bottom panel) samples are compared. Insertions occurring in sense and antisense orientation of gene transcription are marked in red, and green, respectively. (TIF) [file pcbi.1005950.s006.tif]

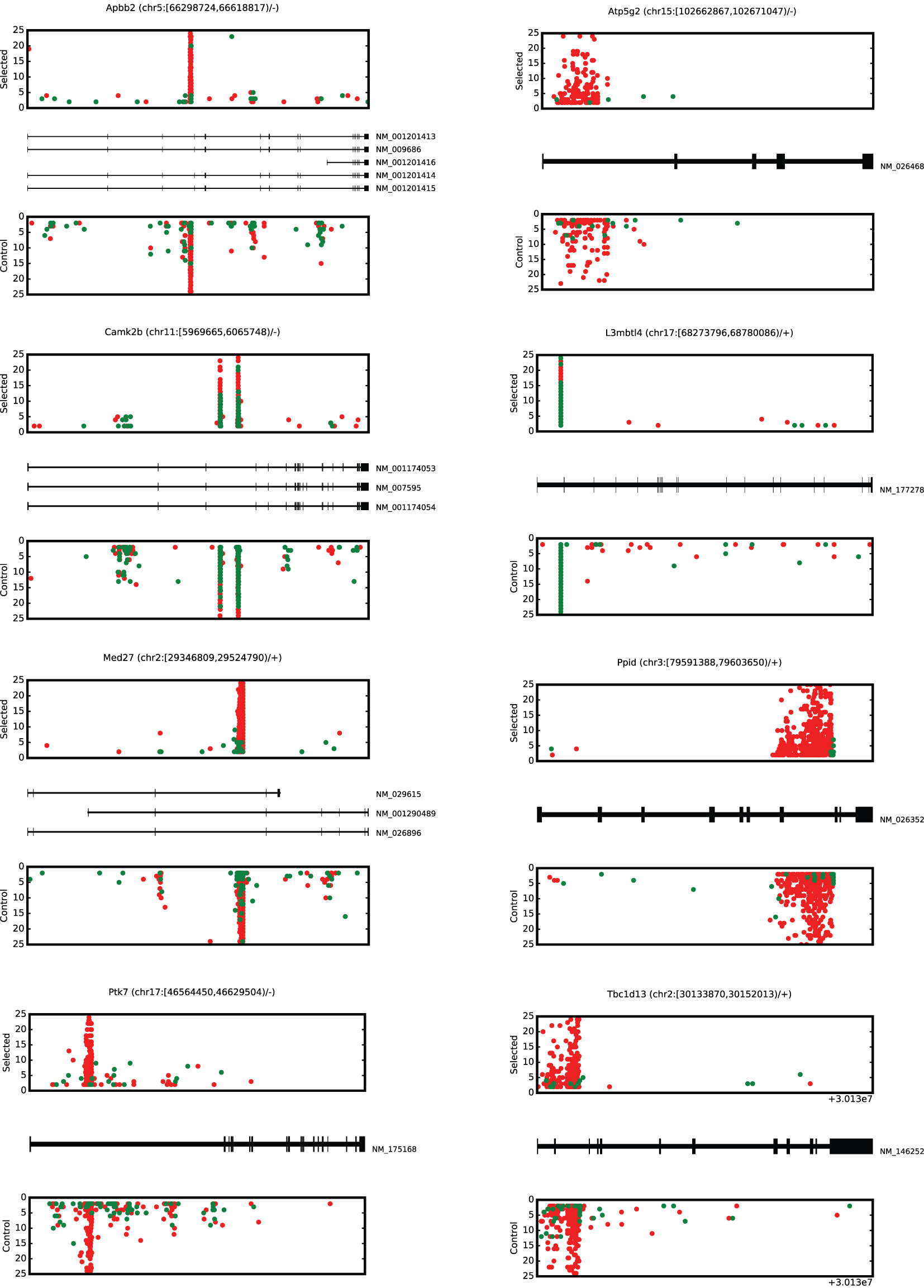

Supplement: S6 Fig — Distribution of I.I. within genes detected by the FT algorithm (Fisher-only). Selected (top panel) and Control (bottom panel) samples are compared. Insertions occurring in sense and antisense orientation of gene transcription are marked in red, and green, respectively. (TIF) [file pcbi.1005950.s007.tif]

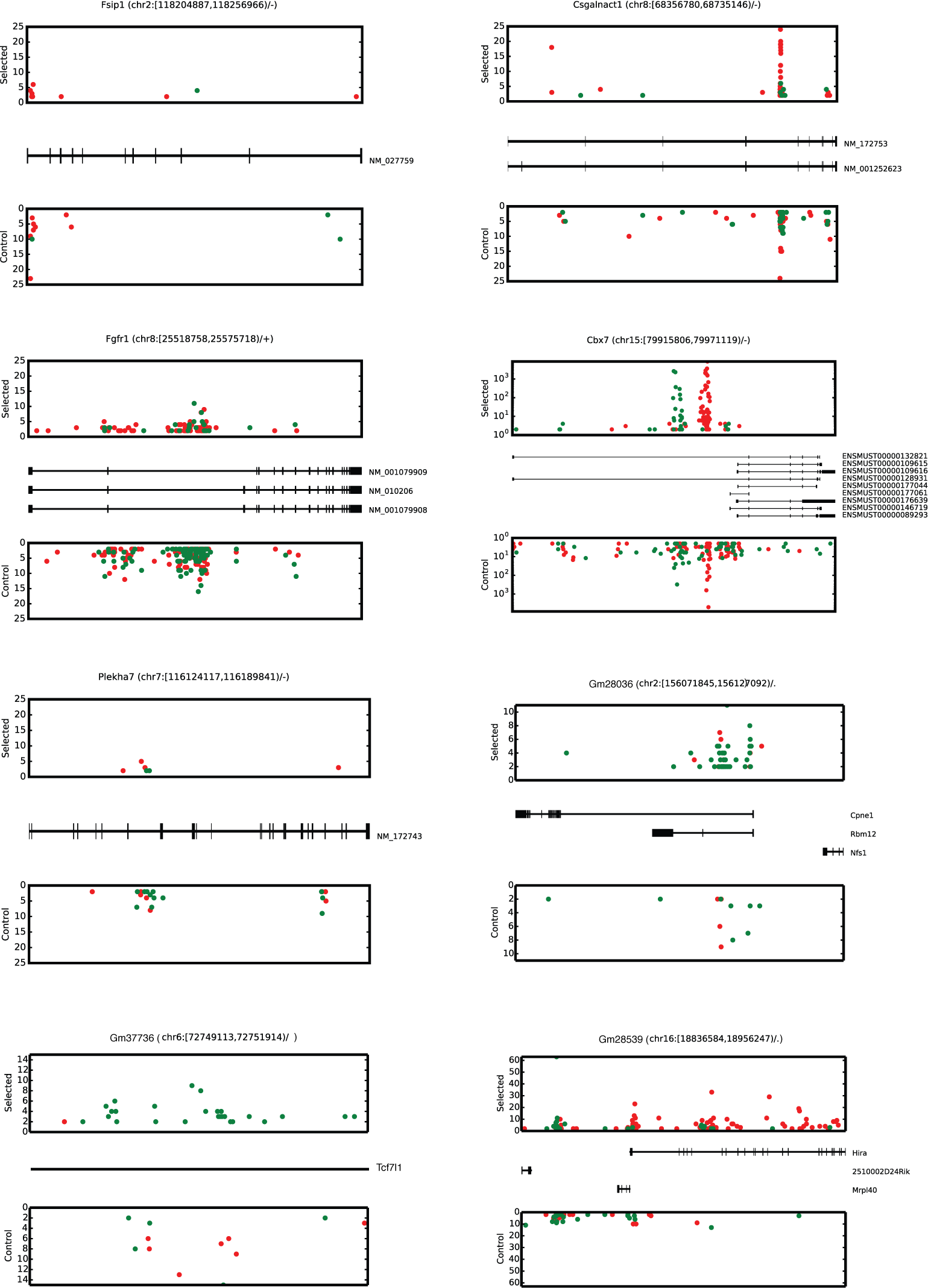

Supplement: S7 Fig — Distribution of I.I. within genes detected by VISITs algorithm (VISITs-only). Selected (top panel) and Control (bottom panel) samples are compared. Insertions occurring in sense and antisense orientation of gene transcription are marked in red, and green, respectively. (TIF) [file pcbi.1005950.s008.tif]

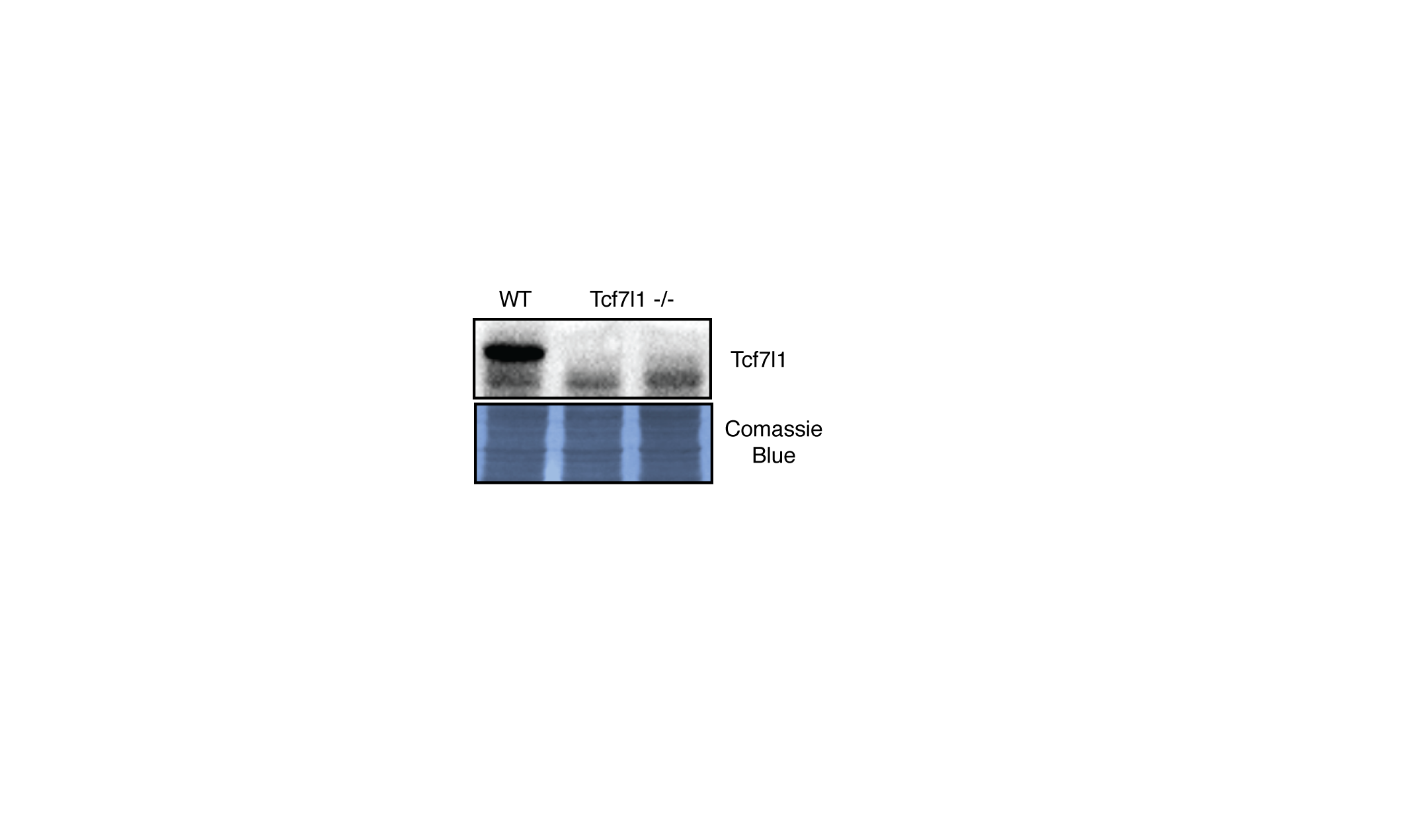

Supplement: S8 Fig — Western analysis of two Tcf7l1 deficient (-/-) and one control (WT) HATX3 ES cell clones. Commassie Blue staining is used to control for loading (below). (TIF) [file pcbi.1005950.s009.tif]

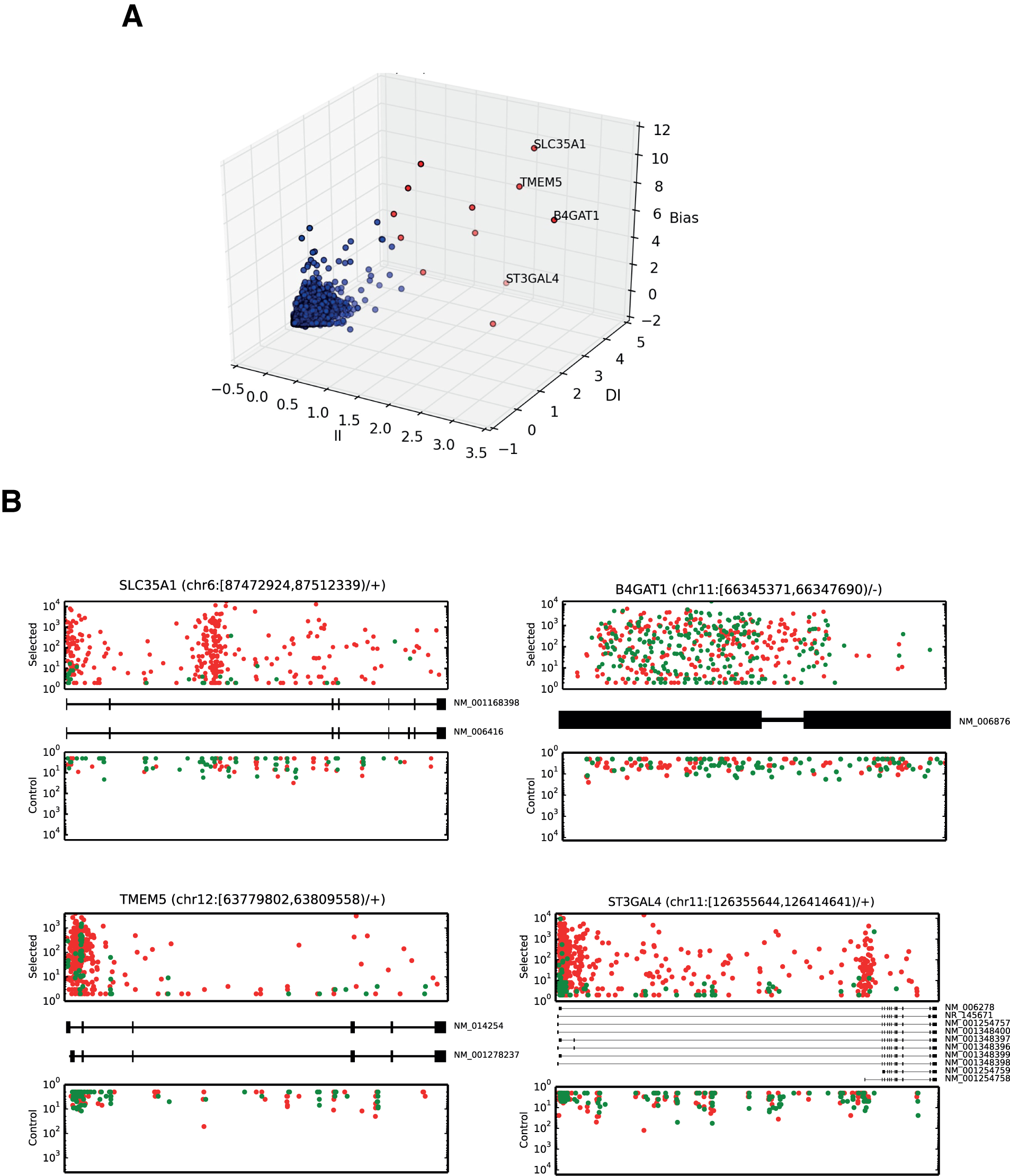

Supplement: S9 Fig — A) Plot of genes represented according to fold enrichment during selection in I.I., D.I. and Bias. The 12 top ranked genes using the LOF algorithm are shown in red. The positions of SLC35A1, B4GAT1, TMEM5, and ST3GAL4 are annotated. (B) Distribution of I.I. at the level of genes detected by HaSAPPy and biologically validated in Jae et al., 2013. Selected (top panel) and Control (bottom panel) samples are compared. Insertions occurring in the orientation of gene transcription are marked in red, anti-sense insertions are marked in green. (TIF) [file pcbi.1005950.s010.tif]

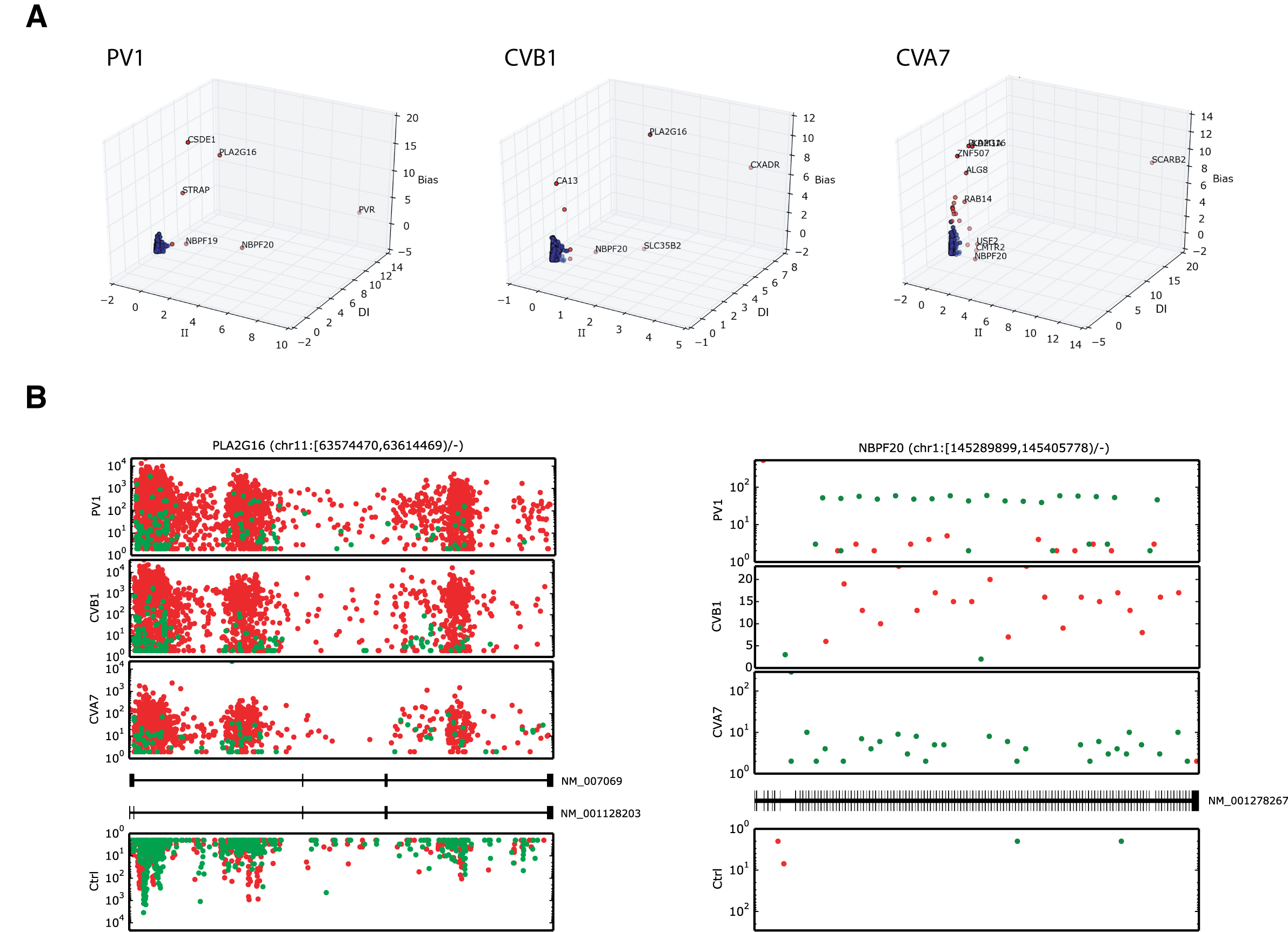

Supplement: S10 Fig — (A) Plot of genes represented according to fold enrichment during selection in I.I., D.I. and Bias for Poliovirus (PV1), Coxsackievirus B1 (CVB1) and Coxsackievirus A7 (CVA7) infection. Genes characterized by a LOF value higher than 15 are shown in red. Genes with a LOF value higher than 20 are annotated in the plot. (B) Distribution of I.I. within genes detected by the LOF algorithm and in common among the different selection strategies. PLA2G16 was biologically validated in Staring et al., 2017. Selected (PV1, CVB1 and CVA7—top panels) and Control (bottom panel) samples are compared. Insertions occurring in sense and antisense orientation of gene transcription are marked in red, and green, respectively. (TIF) [file pcbi.1005950.s011.tif]
